# Research Review: Omega‐3 supplementation to reduce antisocial behavior – a systematic review and meta‐analysis of randomized controlled trials

**DOI:** 10.1111/jcpp.70153

**Published:** 2026-04-14

**Authors:** Adrian Raine, Helena Q. Saven

**Affiliations:** ^1^ Departments of Criminology, Psychiatry, and Psychology University of Pennsylvania Philadelphia PA USA; ^2^ Department of Psychology University of Pennsylvania Philadelphia PA USA

**Keywords:** Omega‐3, antisocial, externalizing, nutrition, randomized controlled trial, meta‐analysis

## Abstract

**Background:**

Omega‐3, a long‐chain fatty acid critical for brain structure and function, has been argued to be effective in reducing antisocial behavior, but findings are variable. This meta‐analysis examines 25 randomized controlled trials (RCTs) with a total of 2,889 participants.

**Methods:**

This study was registered with PROSPERO (CRD42021256959). Two separate analyses were conducted where the unit of analysis was independent studies and independent laboratories.

**Results:**

Significant effect sizes (*p* < .001) were observed for both analyses (*g* = .13, .14 respectively) and in the direction of omega‐3 supplementation reducing antisocial behavior. There was equivocal evidence of publication bias. Study quality was good. Stratified analyses were nonsignificant for age, gender, dose, sample size, and study quality, although the effect size was larger for unmedicated participants. Combining these data with a recent meta‐analysis on aggressive behavior produced an overall effect size of *g* = .22 for externalizing behavior based on 36 nonoverlapping RCTs.

**Conclusions:**

Given that omega‐3 supplementation is relatively cheap, easy to implement, transfers well from the research environment to community settings, and has additional health benefits, it is suggested that it may be considered as an adjunctive intervention for the treatment of antisocial and externalizing behavior.

## Introduction

There is increasing interest in the nascent field of ‘nutritional psychology’, which can be broadly defined as the examination of relationships between nutritional intake and psychological health and well‐being. At a general level, reviews have argued that diet plays a significant role in mental health functioning (Grajek et al., 2022). At a more specific level, it has been argued that micronutrients play a foundational role in maintaining good mental health functioning (Rucklidge, Johnstone, & Kaplan, 2021). Omega‐3 is another component of the diet which has been argued to play a significant role in mental health (Bozzatello, De Rosa, Rocca, & Bellino, 2020). Some randomized controlled trials (RCTs) have suggested that dietary supplementation with this long‐chain fatty acid may help reduce psychopathology. One pioneering RCT that gave omega‐3 supplements to teenagers with subthreshold psychosis documented a reduced transition to psychosis (Amminger et al., 2010). While a later study failed to replicate this finding in ultra‐high‐risk teenagers (McGorry et al., 2017), another RCT found that omega‐3 supplementation reduces schizotypal personality in children (Raine et al., 2024). Reviews of other psychopathologies have been equally mixed, with some arguing for efficacy for depression (Liao et al., 2019), while others argue that the evidence to date is either conflicting (Deacon, Kettle, Hayes, Dennis, & Tucci, 2017) or insufficient (Appleton et al., 2021).

Another condition which may benefit from omega‐3 supplementation consists of antisocial behavior. Poor nutritional status in the first 3 years of life in 1,206 children has been associated with later externalizing behavior at ages 8, 11, and 17 years (Liu, Raine, Venables, Dalais, & Mednick, 2004), suggesting that nutrition per se may play some role in this behavior problem. In this same sample, an experimental intervention that included better nutrition from ages 3–5 years was associated with reduced antisocial behavior at age 17 years and reduced criminal behavior at age 23 years (Raine, Mellingen, Liu, Venables, & Mednick, 2003). Beneficial effects of the intervention were significantly greater for children with malnutrition at age 3 which suggests, but does not prove, that the nutritional component of the intervention may account for some of the improvement. Because the children in the intervention received over two child portions of fish per week more than the control group (Venables & Raine, 2012), we had hypothesized that omega‐3 could be the active ingredient in the enrichment that helped to reduce later antisocial behavior (Raine et al., 2003).

The strongest evidence for a causal relationship between omega‐3 supplementation and reduced antisocial behavior emanates from RCTs. A number of reviews have argued affirmatively for the potential beneficial effects of this intervention on this behavior problem, suggesting that the link between poor nutrition and antisocial behavior could be explained by omega‐3 (Choy & Raine, 2018; Cooper, Tye, Kuntsi, Vassos, & Asherson, 2016; Gow & Hibbeln, 2014). However, these reviews are either not systematic, or they are limited in scope to just eight studies. There appears to be variability in findings of RCTs, with some finding positive effects (Richardson & Montgomery, 2005), some finding null effects (Crippa et al., 2019), and some finding negative effects (Bos et al., 2015). As such, it remains unclear whether omega‐3 supplementation significantly reduces antisocial behavior. The importance of this issue lies in the major economic and health costs of antisocial behavior. One longitudinal study of antisocial children found that, compared with controls, those on the antisocial life‐course path had three times the level of emergency hospital visits, 3.6 times the number of prescription fills, twice the number of hospital bed nights, and 6.5 times the number of social welfare benefits (Rivenbark et al., 2018). While only accounting for 9% of the total sample, they accounted for 53.3% of all criminal convictions, an outcome which in turn evokes a high financial and health care cost to victims and society alike.

Against this backdrop, this study employs meta‐analysis to more systematically evaluate whether there is, or is not, a significant effect of omega‐3 supplementation on antisocial behavior. Because we recently conducted a meta‐analysis specifically of aggressive behavior (Raine & Brodrick, 2024), the current analysis focused on generic antisocial behavior, excluding findings on purely aggressive behavior. In addition to computing an effect size where the unit of analysis is independent samples, a second analysis was conducted to assess the robustness of findings in which the unit of analysis was independent laboratories; effect sizes from several studies derived from the same laboratory were averaged, thus preventing a few laboratories from overly influencing the overall effect size. To examine the variability that we have observed in prior findings in our own and others' research, we examined variables that could change the overall effect. Do any effects of supplementation apply equally to both genders and across all ages, and are effects influenced by whether participants are already receiving medication? We then merge the new antisocial data set with our recent meta‐analysis of aggression to examine whether treatment effects differ significantly between these two forms of behavior problem. We then combine these data sets to compute an overall effect size for externalizing (antisocial and aggressive) behavior.

## Methods

### Study protocol registration

The study protocol was registered on 30th December 2024 with the International Prospective Register of Systematic Reviews (PROSPERO – https://www.crd.york.ac.uk/PROSPERO/home) prior to data collection (CRD42024591831). This study is written in compliance with the recommendations of the Preferred Reporting Items for Systematic Reviews and Meta‐Analyses (PRISMA) statement (Page et al., 2021). The PRISMA checklist is included in Table S1.

### Inclusion criteria

Studies were included in the meta‐analysis if they met the following inclusion criteria: (1) consisted of an RCT, (2) explicitly measured antisocial behavior, (3) provided supplementation with omega‐3, (4) were conducted on humans, (5) provided sufficient information to compute an effect size, (6) were published in English. We excluded studies involving prenatal supplementation to the mother during pregnancy and effects on the offspring, as well as findings with aggression as the only outcome. We did not exclude studies that also included additional nutritional supplements (e.g. multivitamins) on top of omega‐3, but we examined this as a potential effect modifier.

### Search strategy

Searches were conducted in five databases as follows: PubMed, PsychINFO, Medline, Web of Science, and Scopus. There were no limits on publication date, country, and age to include as many eligible studies as possible. Two authors working independently extracted the data according to a predetermined list of interests. Discrepancies were resolved by consensus. Searches were completed on August 8, 2025. A list of potentially relevant studies was produced for a later evaluation for meeting inclusion criteria.

Search terms consisted of the following: (Docosahexaenoic OR Eicosapentaenoic OR Docosapentaenoic OR alpha‐linolenic acid OR Omega‐3 OR ‘fish oil’ OR DHA OR EPA OR DHA OR ALA) AND (antisocial or ‘conduct disord*’ or oppositional or externalizing or ‘behav* problem*’ or crim* or prison* or psychopath* or callous or ‘disruptive behav*’) AND (‘randomi*ed controlled trial’ or RCT or trial or random* or interven*). In addition, we checked reference sections of articles included in the meta‐analysis in addition to other reviews for links to other relevant articles.

### Outcome measure

The search was focused on antisocial behavior. All studies measured rule‐breaking, oppositional behavior, conduct problems, or minor prison rule violations. Full details of outcome measures for each study are given in Table S2. Aggression measures (e.g. reactive‐proactive aggression, point subtraction task) were excluded as a meta‐analysis on aggression has been recently published (Raine & Brodrick, 2024). Externalizing behavior was not included as it frequently overlaps with aggression; for example, in the commonly used Child Behavior Checklist, externalizing behavior is based on the combination of an aggression scale and a rule‐breaking scale (Achenbach & Rescorla, 2001). We also excluded studies assessing mood, suicide, self‐harm, emotional lability, irritability, and affect dysregulation, as well as personality features associated with antisocial behavior such as impulsivity and narcissism which do not directly reflect antisocial behavior.

### Data extraction

Means, *SD*s, and sample sizes were extracted from each study. In the large majority of cases (20 out of 25), effect sizes were based on group mean differences and standard deviations (*SD*) at baseline and end‐of‐treatment. Other studies either reported the effect size, provided interaction effects from ANOVA designs, or reported % change from baseline where we back‐calculated the *SE* from *p*‐values and sample sizes (Cumpston, McKenzie, Welch, & Brennan, 2022). If *SD*s were missing, we applied methods outlined in the Cochrane Handbook for Systematic Reviews of Interventions to estimate *SD*s and calculate effect sizes (Cumpston et al., 2022). Authors were also contacted to ascertain additional information where necessary. The effect size consisted of Hedge's *g* which corrects for bias due to small sample size. A positive effect size indicates a stronger reduction in antisocial behavior in the omega‐3 group compared with controls. For each study, the effect size and its associated standard error were calculated for data entry.

Where several measures of antisocial behavior were reported, effect sizes were calculated for each measure and a meta‐analysis run on these effect sizes to produce an averaged effect size. Multiple effect sizes from several antisocial measures from one study were not entered separately because this creates dependence among effect sizes which in turn results in a violation of standard meta‐analytic models (Lipsey & Wilson, 2001). Instead, an effect size was based on the average of all individual effects. Similarly, when the antisocial measure was completed by two informants (e.g. parent and child), effect sizes were averaged.

The following variables were also extracted as potential effect modifiers for either subgroup (grouping) or meta‐regression (continuous) analyses: clinical versus community sample, medication (yes/no), added multivitamins to omega‐3 supplement (yes/no), age, gender (% females), treatment duration (weeks), omega‐3 dose (g), publication year, sample size, study quality, and analytic design. We did not adopt a hierarchy of instruments to examine as there were 15 different instruments employed across the 25 RCTs, which made it unrealistic to pool these instruments for a stratified analysis.

### Assessment of study quality

The quality of each study in the meta‐analysis was evaluated using the 14‐item NIH Quality Assessment Tool for Controlled Intervention Studies (2013) (https://www.nhlbi.nih.gov/health‐topics/study‐quality‐assessment‐tools). Each study was independently scored by two coders on 14 items covering randomization, blinding, intention‐to‐treat analysis, dropout rate, adherence to intervention, outcome measure validity/reliability, treatment allocation concealment, avoidance of other interventions, baseline parity, outcome prespecified, and statistical power. Scoring details are given in Appendix S1. Inter‐rater reliability was calculated. An overall quality rating for each study, together with a rating for all studies combined, was independently assigned by each rater according to the NIH categories of ‘good’, ‘fair’, or ‘poor’.

### Statistical analyses

We conducted a random‐effects meta‐analysis using SPSS version 29.0.1 because we anticipated significant variability across studies due to differences in age, gender, antisocial measure, dosage, and treatment duration. An inverse variance method was employed to weight studies based on sample size to yield an estimate of the mean effect size. Two‐tailed tests and 95% confidence intervals (CI) were employed. A robustness check was run, with results from independent studies cross‐referenced with independent laboratories.

Heterogeneity was assessed using the *Q* statistic, reflecting observed variation relative to within‐study error, together with *T* ^2^ and *I* ^2^. The prediction interval was used to assess the dispersion of effect sizes (heterogeneity in true effects across studies – Borenstein, Hedges, Higgins, & Rothstein, 2021). Publication bias was evaluated in two ways. First, Egger's regression was calculated, with a significant test indicating potential bias. Second, Duval and Tweedie's trim‐and‐fill test was run, with asymmetry in the funnel plots suggesting publication bias. If bias was detected, new studies were added to the plot and the overall effect size recalculated.

We conducted stratified analyses to probe how effect sizes for omega‐3 supplementation vary as a function of both group and continuous effect modifiers. For continuous effect modifiers, univariate meta‐regression analyses were conducted. The resulting beta value indicates how treatment effectiveness changes as a function of the modifier variable, with a significant coefficient indicating a linear relationship between the effect modifier and treatment efficacy (Page et al., 2021).

## Results

### Study selection

**Figure 1:**
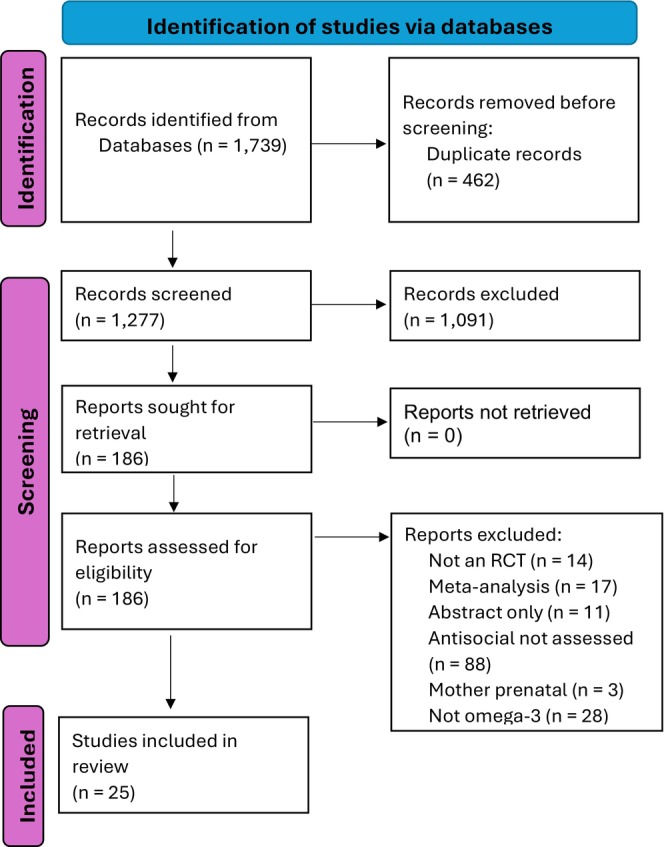
Flow diagram of literature search resulting in the 25 RCT studies included in the omega‐3 – meta‐analysis on antisocial behavior

Full details of the number of records screened and those excluded are given in the flow diagram in Figure 1. Out of 1,739 records screened, 25 published articles met the inclusion criteria, with a total sample of 2,889 participants.

### Characteristics of included studies

Study characteristics are outlined in Table 1. Publication year ranged from 2002 to 2021. Mean age was 10.49 (*SD* 3.41) with the maximum age being 19.50, while female representation was 29.80%. Recruitment was as follows: school (31.8%), clinic (27.3%), community (18.2%), both community and clinic (13.6%), prison (9.1%). Regarding location, the majority of studies were conducted in Europe and North America, with 59.1% conducted in the West, 9.1% in the East, and 31.8% in other geographic areas. Mean duration of supplementation was 17.00 weeks (*SD* = 6.57). Mean omega‐3 dosage was 827 mg (*SD* = 527). Surprisingly, only four authors reported ethnicity, and as such, could not be examined further. Further study characteristics may be found in Table S2.

**Table 1:** Characteristics of studies included in the meta‐analysis

Study	Age (years)	Gender (% female)	Country	Duration (weeks)	Dose (mg)	EPA/DHA content	Recruitment	Quality score	Medication
Bélanger et al. (2009)	9.16	44.0	Canada	16	1,050	EPA + DHA	Clinic	8	Yes
Bos et al. (2015)	10.60	0	Netherlands	16	1,300	EPA + DHA	Clinic/Community	11	Yes
Crippa et al. (2019)	10.98	8.0	Italy	24	500	DHA	Clinic	12	Yes
Dean, Bor, Adam, Bowling, and Bellgrove (2014)	10.3	19.0	Australia	6	2,400	EPA + DHA	Clinic/Community	13	No
Gesch, Hammond, Hampson, Eves, and Crowder (2002)	19.5	0	England	20	124	EPA + DHA + ALA	Prison	13	No
Gustafsson et al. (2010)	9.50	–	Sweden	15	503	EPA + DHA	Clinic	12	No
Johnson, Handen, Zimmer, and Sacco (2010)	3.48	–	USA	12	400	DHA	Clinic	6	No
Kean et al. (2017)	8.70	14.6	Australia	14	45	EPA + DHA	Clinic	12	Yes
Kirby, Woodward, Jackson, Wang, and Crawford (2010)	9.13	52.8	Wales	16	456	EPA + DHA	School	10	No
Manor et al. (2012)	9.20	30.0	Israel	30	120	EPA + DHA	Clinic/Community	13	Yes
Milte et al. (2015)	8.83	27.7	Australia	16	1,256	EPA + DHA	Community	12	No
Raine et al. (2015)	11.32	32.4	Mauritius	24	840	EPA + DHA + ALA	Community	14	No
Raine et al. (2016)	11.46	46.6	USA	12	1,000	EPA + DHA + ALA	Community	12	No
Raine et al. (2019)	10.59	12.4	Singapore	24	1,000	EPA + DHA	Clinic	13	Yes
Raine et al. (2020)	19.25	0	Singapore	12	840	EPA + DHA + ALA	Prison	14	No
Raine et al. (2021)	11.90	32.4	Hong Kong	24	840	EPA + DHA + ALA	School	14	No
Richardson and Puri (2002)	10.25	14.5	Northern Ireland	12	666	EPA + DHA + ALA	School	11	No
Richardson and Montgomery (2005)	8.82	33.3	England	12	732	EPA + DHA	School	14	No
Richardson, Burton, Sewell, Spreckelsen, and Montgomery (2012)	8.69	47.0	England	16	600	DHA	School	14	No
Sinn and Bryan (2007)	9.44	36.0	Australia	15	732	EPA + DHA	Community	8	No
Smuts, Greeff, Kvalsvig, Zimmermann, and Baumgartner (2015)	8.40	50.0	South Africa	34	500	EPA + DHA	School	12	No
Stevens et al. (2003)	9.80	12.2	USA	16	560	EPA + DHA	School	10	Yes
Tammam, Steinsaltz, Bester, Semb‐Andenaes, and Stein (2016)	14.29	50.0	England	12	281	EPA + DHA	School	13	No
Widenhorn‐Muller, Schwanda, Scholz, Spitzer, and Bode (2014)	8.91	22.1	Germany	16	720	EPA + DHA	Clinic	12	No
Young, Arnold, Wolfson, and Fristad (2017)	11.06	61.4	USA	12	2000	EPA + DHA	School	8	Yes

### Study quality

Detailed scores on the NIH Quality Assessment Tool for each study and external validity of the scores are given in Appendix S1, while study quality scores for each study are given in Table 1. Inter‐rater reliability between the two independent evaluators was high, *r* = .92, *N* = 25, *p* < .001. Ratings on each of the 14 items in the assessment tool are given in Figure S1 in Appendix S1. All 25 studies were RCTs. Participant blinding occurred in 88% of studies, while assessor blinding occurred in 96%. Adherence to the treatment regimen was high (96%). All studies used outcome measures with established reliability/validity. The weakest areas consisted of a high dropout rate (48%), while just 48% of studies had adequate power. Eighty‐four percent of the studies were scored as ‘good’, 12% as ‘fair’, and 4% as poor. Overall quality was rated as good.

### Meta‐analytic results and risk of bias

#### Independent samples

**Figure 2:**
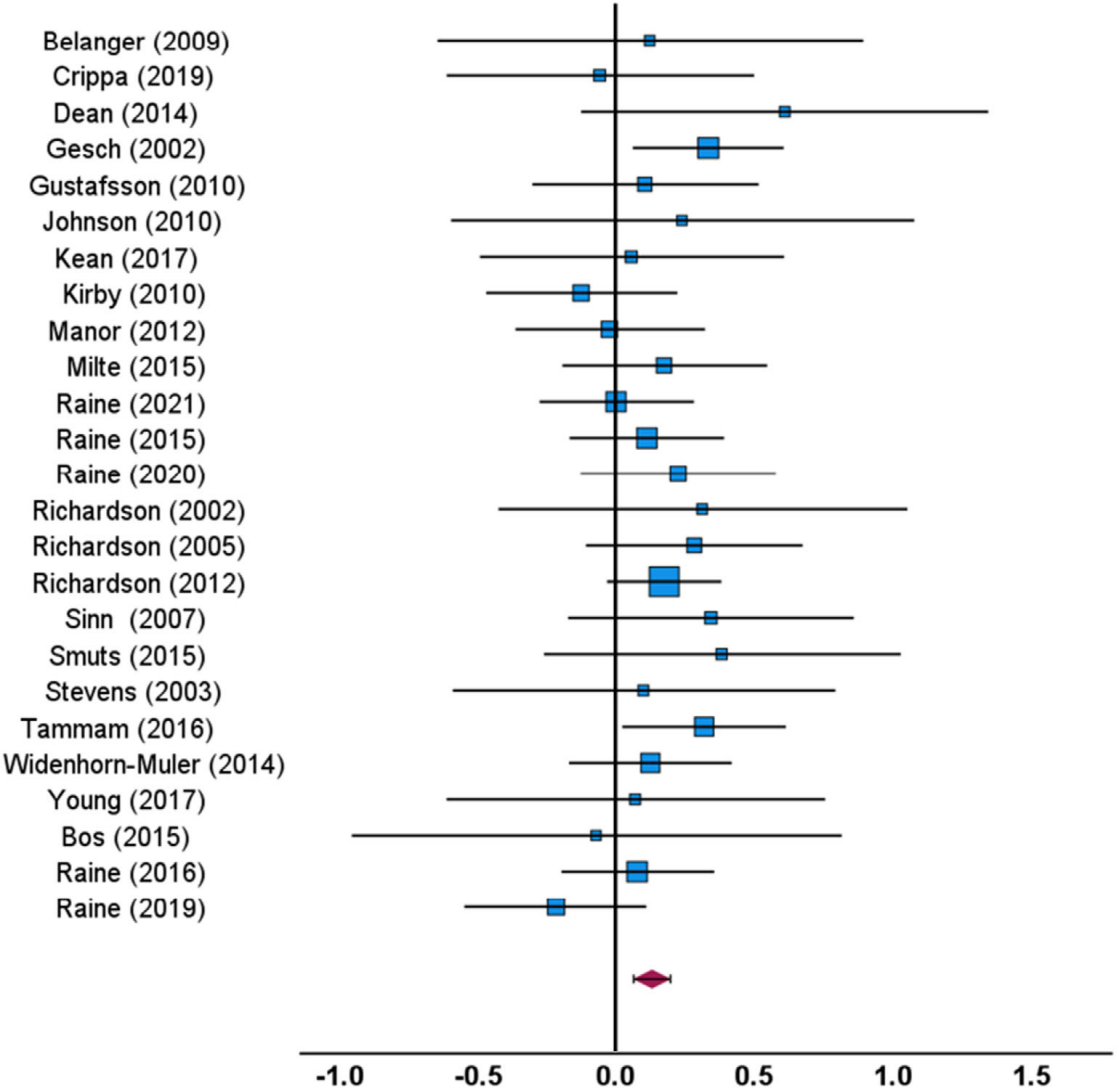
Forest plot of the 28 independent RCT samples. Blue boxes indicate the effect size for each study along with the confidence interval (horizontal lines). The red diamond indicates the overall effect size (*g* = .13) along with its confidence interval. A positive effect indicates that omega‐3 supplementation is associated with reduced antisocial behavior

Effect sizes from the 25 independent RCTs are given in the forest plot in Figure 2. The overall effect size was statistically significant (*g* = .13, *p* = .0005, *SE* = .032, *t* = 4.08, CI = 0.065 to 0.198). The *Q* statistic was nonsignificant (*χ* ^2^ = 11.75, df = 21, *p* = .95). The prediction interval ranged from .065 to .198, with values of 0 for both *T* ^2^ and *I* ^2^.

**Figure 3:**
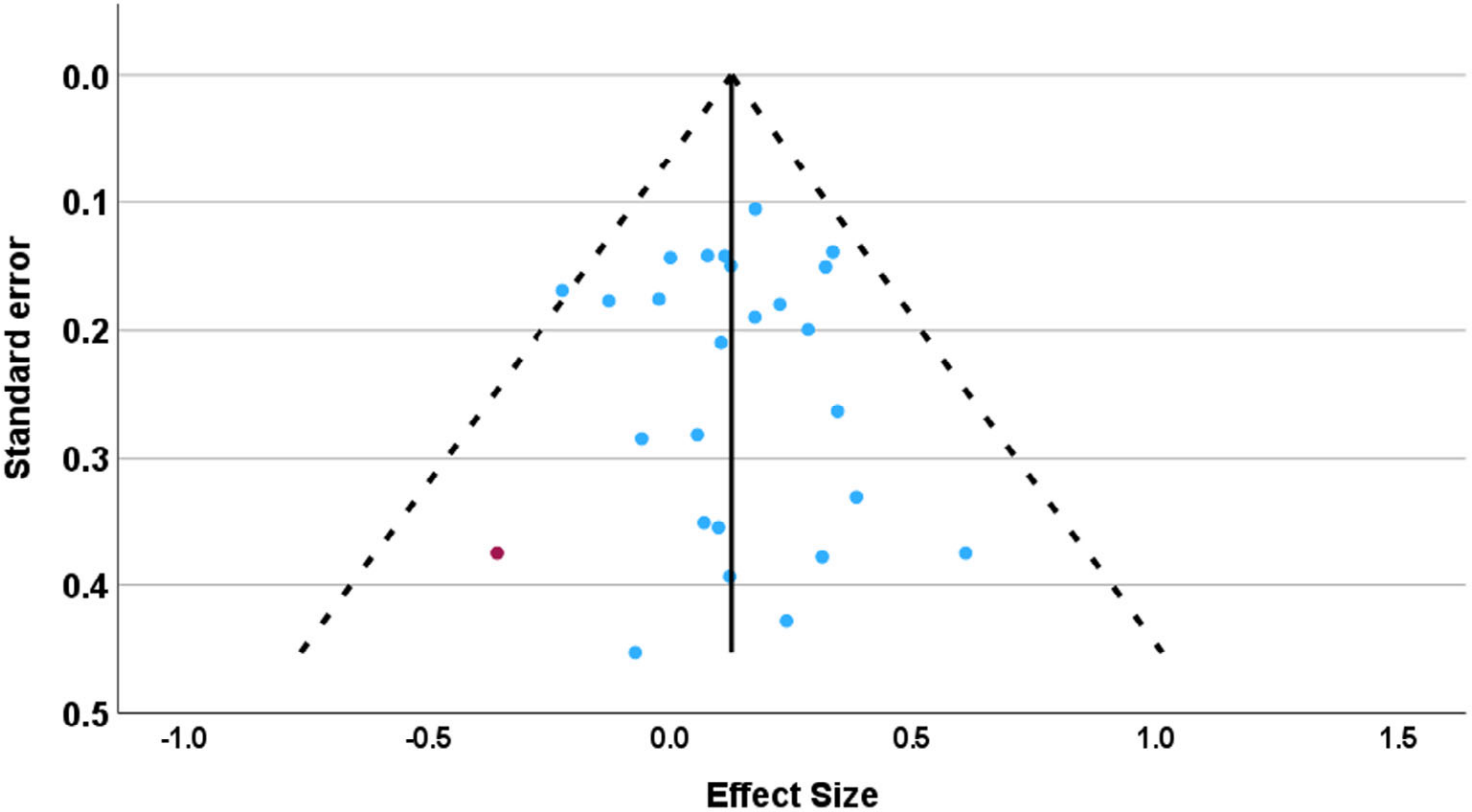
Funnel plot of the effect sizes drawn from the 25 independent RCT studies (blue circles), indicating some evidence of publication bias, with one imputed study (red dot). Dotted lines indicate pseudoconfidence intervals

There was equivocal evidence of publication bias. Egger's regression test was nonsignificant (*p* = .73, intercept = .102, *SE* = .457, *t* = 0.35, CI = −0.783 to 1.107). However, Duval and Tweedie's trim‐and‐fill test indicated some evidence of asymmetry in the funnel plot, with one imputed study changing the effect size slightly from .131 to .126 (see Figure 3).

#### Independent laboratories

Effect sizes from the 19 independent laboratories are given in the forest plot in Figure S2. The overall effect size was statistically significant (*g* = .14, *p* = .0008, *SE* = .034, *t* = 4.02, CI = 0.065 to 0.207). The *Q* statistic was nonsignificant (*χ* ^2^ = 12.92, df = 18, *p* = .79). The prediction interval ranged from .007 to .265, with values of .003 for *T* ^2^ and 7.7% for *I* ^2^.

There was equivocal evidence of publication bias. Egger's regression test was nonsignificant (*p* = .56, intercept = .101, *SE* = .379, *t* = 0.60, CI = −0.574 to 1.024), suggesting no publication bias. Duval and Tweedie's trim‐and‐fill test, however, indicated some evidence of asymmetry in the funnel plot (see Figure S3), with one imputed study changing the effect size from .136 to .130 (*SE* = .035, *t* = 3.72, *p* = .001, CI = 0.057 to 0.203).

### Subgroup and meta‐regression analyses

Not all studies produced an effect size of *g* = .13. The prediction interval showed a spread of .065 to .198, with the forest plot indicating variability in effect sizes ranging from −.22 to .61 (see Figure 2). Because this range can be meaningful, we ran exploratory analyses on the hypothesized effect modifiers in the 25 RCT studies.

Results of categorical (subgroup) and dimensional (meta‐regression) effect modifiers are shown in Table 2. Dimensional effect modifiers were largely nonsignificant with two exceptions: publication year (larger effect sizes for earlier studies) and treatment duration (larger effect size with shorter duration). Two categorical effect modifiers were significant: medication (stronger effect size in unmedicated) and analytic design (stronger effects in per protocol approach). Form of antisocial behavior (ODD, CD, rule‐breaking) did not change treatment outcome (see Appendix S1).

**Table 2:** Results of meta‐regression stratified analyses based on continuous covariates (a) and categorical effect modifiers (b), together with effect sizes (Hedge's *g*) for subgroups

(a)					
Covariate	*β*	*SE*	*t*	*p*	CI
Age	.016	.009	1.82	.081	−0.268 to 0.165
Gender	−.001	.002	−0.29	.773	−0.005 to 0.003
**Treatment duration**	−.012	.005	−2.23	.036	−0.023 to −0.001
Omega‐3 dose	.000	.000	−0.66	.519	0.000 to 0.000
Sample size	.000	.000	−0.48	.639	−0.001 to 0.001
Study quality	−.032	.022	−1.47	.156	−0.077 to 0.013
**Publication year**	−.014	.006	−2.44	.023	−0.025 to −0.002

(b)								
Effect modifier	*F*	*p*	Subgroup	Effect size (*g*)	*SE*	*t*	*p*	CI
Clinical vs. Community	0.52	.479	Clinical	.18	.061	0.399	.0007	0.091 to 0.263
			Community	.13	.074	0.046	.019	0.028 to 0.238
**Medication**	11.95	.009	Unmedicated	.17	.034	5.285	.0002	0.099 to 0.245
			Medicated	−.03	.047	−0.602	.566	−0.144 to 0.087
**Analytic design**	4.50	.045	ITT	.10	.040	0.039	.022	0.016 to 0.180
			PP	.25	.037	7.137	.0002	0.159 to 0.353
Multivitamins	0.13	.718	Added	.14	.091	1.55	.196	−0.112 to 0.395
			Not added	.12	.037	3.022	.004	0.056 to 0.195

Significant covariates/modifiers are highlighted in bold.

CI, confidence interval; ITT, intention to treat; PP, per protocol; *SE*, standard error.

### Antisocial behavior and aggressive behavior

After combining the current antisocial behavior data with our prior meta‐analysis of 29 RCTs on aggression, a stratified analysis was conducted to evaluate whether the effect size for antisocial behavior differed from the effect size for aggressive behavior (Raine & Brodrick, 2024). Because some studies contributed to both forms of disruptive behavior and would violate the independence assumption, we selected the 36 RCTs that were independent of one another (20 for aggression, 16 for antisocial behavior). Effect sizes for the two groups were as follows: aggression (*g* = .31, *SE* = .112, *t* = 2.74, *p* = .014); antisocial (*g* = .17, *SE* = .036, *t* = 4.82, *p* = .0002). The stratified analysis was nonsignificant (*F* = 0.90, df = 1,34, *p* = .35), indicating no significant difference between the two forms of disruptive behavior problems.

An analysis was run to compute the effect size for externalizing behavior more broadly (antisocial and aggression studies combined) based on the above 36 RCTs. The overall effect size was significant (*g* = .22, *SE* = .056, *t* = 3.991, *p* = .0003, CI = 0.109 to 0.336, prediction interval = −.218 to .663, *T* ^2^ = .044, *I* ^2^ = 49.8%).

## Discussion

This meta‐analysis set out to investigate the effect of omega‐3 supplementation on antisocial behavior using two main units of analysis. Analyses from 25 RCTs were based on 28 independent samples with a total sample of 2,889 participants, and a second analysis focused on independent laboratories. Overall effect sizes for the two analyses were as follows: independent studies (*g* = .13) and independent laboratories (*g* = .14). There was equivocal, limited evidence of publication bias. Exploratory stratified analyses suggested that treatment duration, publication year, medication, and analytic design may influence findings. Effects for age and gender were nonsignificant, suggesting at a study level that omega‐3 supplementation may be helpful to populations of all ages and genders. In both analyses for antisocial behavior the prediction intervals were in positive territory and did not cross 0, giving assurance that supplementation in the context of true effect sizes will be positive and not negative at least 95% of the time. Overall study quality was good. Combining this meta‐analysis with a prior meta‐analysis on aggression yielded an effect size of *g* = .22 for externalizing behavior. Results provide some evidence that omega‐3 supplementation can lead to modest short‐term reductions in antisocial behavior.

### Mechanisms‐of‐action

Why would omega‐3 supplementation be expected to reduce antisocial behavior? At a mechanistic level, it is known that omega‐3 is a long‐chain fatty acid that plays a critical role in brain structure and function, and one specific mechanism of action may lie with respect to the prefrontal cortex. Concentration levels of DHA vary throughout the brain and are at their highest in the prefrontal cortex (Laye, Nadjar, Joffre, & Bazinet, 2018), an area critical for impulse control and emotion regulation. Higher levels of omega‐3 are associated inter alia with increased functional connectivity in the frontal pole and anterior cingulate, areas that subserve executive functions (Talukdar, Zannroziewicz, Zwilling, & Barbey, 2019). Omega‐3 supplementation has also been shown to enhance executive functions (McNamara et al., 2010), and importantly poor executive functioning is a well‐replicated risk factor for antisocial behavior (Jansen & Franse, 2024). More broadly, omega‐3 has wide‐ranging effects on the brain, making up ~ 35% of the cell membrane, enhancing neurite outgrowth, regulating both neurotransmitter functioning and gene expression, and being involved in neurogenesis and nerve cell signaling (McNamara & Carlson, 2006; Bazinet & Laye, 2014; Diaz, Mesa‐Herrera, & Marin, 2021). Given that brain imaging research has shown that antisocial individuals have wide‐ranging impairments to brain structure and function (Carlisi et al., 2020; Tully et al., 2023), it is reasonable to anticipate that omega‐3 supplementation, with its widespread effects on the brain, can be hypothesized to play a causal role in reducing antisocial behavior by upregulating the brain.

### Effect modifiers

Although study findings were not found to be heterogenous as indicated by a nonsignificant *Q* statistic (*p* = .95), effect sizes varied somewhat from −.22 to .61, suggesting some clinical variation in study outcomes. The prediction interval range was narrower, but still indicated some variability in true effects. We therefore conducted exploratory analyses to probe this variation in true effect sizes.

The majority of effect modifiers were nonsignificant. In particular, age and gender did not influence the efficacy of treatment, suggesting that at a study level, supplementation can benefit populations of both male and female children equally, as well as children of all ages, at least within the range encompassed by the current RCTs (3.5–19.5 years). Sample size and study quality were not associated with effects, suggesting that the obtained effect size was not attributable to poorer quality studies. Dosage was unrelated to outcome, suggesting that even modest levels of omega‐3 supplementation may be beneficial.

Stratified analyses did however reveal some significant findings. As commonly observed in other meta‐analyses, stronger effects were observed in earlier studies, suggesting the possibility that the effect size may further reduce over time. An unexpected result was that stronger effects were observed in RCTs with a shorter treatment duration. Analytic design had an influence, with intention‐to‐treat designs producing weaker effects. This could suggest that this design, which utilizes all randomized participants regardless of completion, provides a more unbiased estimate of the true effect size than per protocol analyses, which focus only on those who complete treatment. Alternatively, per protocol analyses may produce stronger effects because of the very fact that treatment regimen has been maximized. In addition, medication was a significant effect modifier, with children receiving medication showing a null effect (*g* = −.03, *p* = .57) compared with those not medicated (*g* = .17, *p* = .0002). It is conceivable that the medication children receive for their disorder reduces antisocial behavior to a level which places a floor effect on omega‐3 efficacy. For example, 10 of the RCTs were conducted on children with ADHD where stimulants are known to reduce disruptive behavior problems, with an effect size of 0.63 (Seok et al., 2023). These findings are suggestive and could guide further hypothesis‐testing in future RCTs.

### Effects for aggressive versus antisocial behavior, and overall externalizing behavior

We utilized our recent omega‐3 meta‐analysis on aggression (Raine & Brodrick, 2024) to ascertain whether supplementation was more effective for one form of disruptive behavior problems than the other. The moderation analysis was nonsignificant (*p* = .35), suggesting that omega‐3 is equally effective in reducing both forms of disruptive behavior problems. We caution, however, that the effect for aggression (*g* = .31) was almost twice as high as that for antisocial behavior (*g* = .17), and that with more studies in the future there is the possibility for this difference to become statistically significant. Taken together, 36 nonoverlapping RCT studies on aggression and antisocial behavior lead to a collectively larger body of evidence, documenting an average effect size of *g* = .22 for disruptive behavior problems. More broadly, therefore, there is stronger evidence to support omega‐3 supplementation for treating externalizing behavior problems, although this overall effect is still modest in size.

### Recommendations for future research

Based on a more qualitative analysis of the studies making up this meta‐analysis, we have several recommendations for future research. With respect to population‐based parameters, there are virtually no studies on adult antisocial behavior. Mean participant age overall was 10.49 years, with only two studies (both on young offenders) evaluating antisocial behavior in young adults (mean ages 19.25 and 19.5 years). Females were underrepresented, constituting just 27.5% of participants. Only four studies reported ethnicity, negating the ability to evaluate the effect of this demographic on treatment outcome. Type of medication used was underreported and more detailed reporting in future studies would be desirable. One antisocial group of note that could not be analyzed constitutes individuals with psychopathy. There were two reasons for this omission. First, only one laboratory assessed psychopathic personality. Second, the measures used were impulsivity, narcissism, and callous‐unemotional traits, personality correlates of antisocial behavior that we specifically excluded as they do not directly reflect antisocial behavior (see Methods). A further issue is that while studies on ADHD populations were well‐represented, studies on conduct disorder and oppositional defiant disorder were underrepresented, and yet these constitute very pertinent populations. Given these considerations we recommend that future studies on adults, females, children with disruptive behavior disorders, and studies employing behavioral measures of psychopathy would help fill these significant gaps, while future studies should report ethnicity in order that this demographic can be more thoroughly evaluated.

Regarding methodology, one important neglected issue concerns longer term effects of omega‐3 supplementation on antisocial behavior, given that only one laboratory has followed up participants after treatment is terminated. Long‐term beneficial effects 6 months post‐treatment (*d* = .59) for child externalizing behavior have been reported in one study from Mauritius (Raine, Portnoy, Liu, Mahoomed, & Hibbeln, 2015), while another RCT from the USA observed beneficial effects (*d* = .36) 3 months post‐treatment, with a trend for improvement (*d* = .28) 9 months post‐treatment (Raine et al., 2016). Two child RCTs, however, failed to observe long‐term effects in East Asian populations (Singapore and Hong Kong – Raine et al., 2019; Raine, Fung, Gao, & Lee, 2021). This could be due in part to higher rates of fish consumption in these countries producing a ceiling effect (Micha et al., 2014), although in contrast another RCT from Singapore on young offenders demonstrated beneficial effects 3 months (*d* = .39) and 9 months post‐treatment (*d* = .43) (Raine, Leung, Singh, & Kaur, 2020), as well as a trend for reduced recidivism 3 years post‐release (Raine, Choy, Leung, Singh, & Kaur, 2023). Based on these initial findings, it is recommended that future studies move beyond short‐term effects toward conducting longer term tests of the efficacy of omega‐3 supplementation.

Several other methodological issues should be considered. Most studies (64.3%) have employed a single outcome measure. In our experience, we find that measurement based on multiple measures of antisocial behavior produces more robust effects than single measures, likely due to enhanced reliability and content validity (e.g. Raine et al., 2020), an issue that could be probed in future studies. Regarding dosage, we note that the average amount of omega‐3 was 827 mg, which is relatively low given that doses up to 5 g/day are considered safe (EFSA, 2012) and that doses up to 4 g /day are given in studies of major depression (Firth et al., 2019). Future studies could explore whether or not higher doses beyond those used in the current studies may yield potentiated findings. Blood levels of omega‐3 supplementation before and after treatment are rarely taken, but these are important to validate compliance with the intervention. No study has examined the moderating effects of genetic makeup; for example, the FADS gene cluster is one variant known to significantly impact omega‐3 biosynthesis, and is argued to be a major factor in the heterogeneity of clinical trials of omega‐3 and heart disease (Fernandez et al., 2021). Finally, as alluded to earlier, it is recommended that future studies probe putative mechanisms‐of‐action to better understand exactly *why* omega‐3 reduces antisocial and externalizing behavior.

### Limitations

Limitations of the current study need to be recognized. Results of stratified analyses need to be treated cautiously and as giving pointers only for future studies. While findings are based on 25 RCTs, and while this is a reasonable number for health‐related RCT meta‐analyses, it places limits on both overall findings and stratified analyses, which are vulnerable to Type 2 error. Only studies published in English were analyzed, and insufficient reporting resulted in our inability to evaluate the influence of ethnicity, an important issue that needs to be addressed in future studies. Findings on children cannot be extrapolated to adults beyond the two studies of young offenders.

## Conclusions and clinical significance

Limitations notwithstanding, this is the first meta‐analysis of omega‐3 supplementation to reduce antisocial behavior, indicating an average effect size of *g* = .135. It also constitutes the first meta‐analysis of externalizing behavior, with an effect size of *g* = .22. We underline the fact that the overall effect sizes reported here for antisocial behavior in particular and externalizing behavior in general are modest. From a clinical standpoint, these effects would translate to estimated odds ratios of 0.78 and 0.67 respectively, suggesting a 22% reduction in the probability of antisocial behavior and 33% for externalizing behavior. Clearly omega‐3 supplementation is not a panacea for these disruptive behavior problems, and other interventions such as parent‐training, CBT, and pharmacological interventions show stronger efficacy effects (Beelmann, Arnold, & Hercher, 2023; Pezzoli et al., 2024; Seok et al., 2023).

At the same time, there are reasons to consider omega‐3 supplementation for antisocial behavior. The fact that omega‐3 was found to be more effective in those not receiving medication could be of particular interest to healthcare professions treating antisocial children outside of the clinic and to those families in the community who prefer nonpharmacological options for treating antisocial behavior. While some other interventions are more effective, there are practical issues in the ability to move these programs from the high‐fidelity, controlled research environment into clinical practice (Baskin‐Sommers et al., 2024). In contrast omega‐3 supplementation is easy to implement and is a relatively cheap intervention. Unlike other interventions, supplements are not necessary to implement change; caregivers advised to add just one 3 oz portion of fish (e.g. salmon) to the weekly diet (yielding 1.830 mg of EPA and DHA), would result in a fivefold increase to the weekly intake of EPA and DHA for children and adolescents which is estimated at just 333 mg and 414 mg respectively (National Heart, Lung and Blood Institute, 2013; NIH, 2023; Thompson et al., 2019; USDA, 2015). When other interventions are not effective, omega‐3 could be considered as an alternative. Omega‐3 supplementation is also considered safe and could be used as an alternative to medication. Omega‐3 has been shown to have a number of additional health benefits, including cognitive functioning in youths (Emery et al., 2020), childhood sleep health (Dai & Liu, 2021), and depression (Kelaiditis, Gibson, & Dyall, 2023). These factors could be taken into consideration when considering whether to use omega‐3 supplementation as an adjunctive intervention for disruptive behavior problems.

## Ethical considerations

Not applicable: no human subjects participation.

## Trial registration

The study protocol was registered on 30th December 2024 with the International Prospective Register of Systematic Reviews (PROSPERO – https://www.crd.york.ac.uk/PROSPERO/view/CRD42024591831) prior to data collection (CRD42024591831).

**Key points**

**What's known?**

- Omega‐3 is known to be critical to brain structure and function, but does it help reduce antisocial and externalizing behavior?

**What's new?**

- We reviewed 25 randomized controlled trials which had examined the effect of omega‐3 supplementation on antisocial behavior. Overall, we found that omega‐3 reduced antisocial behavior by ~22%, and that it reduced externalizing behavior by ~33%.

**What's relevant?**

- While omega‐3 will not replace other treatments which produce stronger effect sizes, in clinical practice it could yield beneficial effects when used as an adjunct to other interventions.

## Supporting information


**Table S1.** PRISMA checklist.
**Table S2.** Further details of the RCT studies contained in the meta‐analysis. Acronyms are as follows: ALA, alpha‐linolenic acid; ASR, Adult Self‐Report (Achenbach & Rescorla, 2001); ABCL, Adult Behavior Checklist (Achenbach & Rescorla, 2001); CBCL, Child Behavior Checklist (Achenbach & Rescorla, 2001); CD, conduct disorder; CODDS, Conduct and Oppositional defiant Disorder Scales (Raine et al., 2022); DISC‐IV, NIMH Diagnostic Interview Schedule for Children, Version IV (Schaffer et al., 2000); ODD, oppositional defiant disorder; DHA, docosahexaenoic acid; EPA, eicosapentaenoic acid; SDQ, Strengths and Difficulties Questionnaire (Goodman, 1997); SNAP‐IV, Swanson, Nolan and Pelham – IV (Parent‐reported rating scale) (Swanson, 1992).
**Appendix S1.** Study quality assessment.
**Figure S1.** Consensus evaluations on each of the 14 items in the Quality Assessment Tool.
**Figure S2.** Forest plot of the 19 independent laboratories. Blue boxes indicate the effect size for each study along with the confidence interval (horizontal lines). The red diamond indicates the overall effect size (*g* = .162) along with its confidence interval. A positive effect indicates that omega‐3 supplementation is associated with reduced antisocial behavior.
**Figure S3.** Funnel plot of the effect sizes drawn from the 19 independent laboratories (blue circles), with one imputed study (red dot). Dotted lines indicate pseudo confidence intervals.

## Data Availability

Data are available from the first author upon reasonable request.
