# Supplementary material for: Research Review: Omega‐3 supplementation to reduce antisocial behavior – a systematic review and meta‐analysis of randomized controlled trials
Source: J Child Psychol Psychiatry. 2026 Apr 14;67(9):1601–13. doi: 10.1111/jcpp.70153 (PMC13450281; doi:10.1111/jcpp.70153)
Supplement: Supplementary file 1 [file JCPP-67-1601-s001.docx]

**Omega-3 Supplementation to Reduce Antisocial Behavior: A Systematic Review and Meta-Analysis of Randomized Controlled Trials**

**Supporting Information**

**Table S1** PRISMA checklist

| **Section and Topic** | **Item #** | **Checklist item** | **Location where item is reported** |
| --- | --- | --- | --- |
| **TITLE** | | |  |
| Title | 1 | Identify the report as a systematic review. | 1, 2,4 |
| **ABSTRACT** | | |  |
| Abstract | 2 | See the PRISMA 2020 for Abstracts checklist. | 2 |
| **INTRODUCTION** | | |  |
| Rationale | 3 | Describe the rationale for the review in the context of existing knowledge. | 3-5 |
| Objectives **Table 1. PRISMA checklist** | 4 | Provide an explicit statement of the objective(s) or question(s) the review addresses. | 4-5 |
| **METHODS** | | |  |
| Eligibility criteria | 5 | Specify the inclusion and exclusion criteria for the review and how studies were grouped for the syntheses. | 5-6 |
| Information sources | 6 | Specify all databases, registers, websites, organisations, reference lists and other sources searched or consulted to identify studies. Specify the date when each source was last searched or consulted. | 6 |
| Search strategy | 7 | Present the full search strategies for all databases, registers and websites, including any filters and limits used. | 6 |
| Selection process | 8 | Specify the methods used to decide whether a study met the inclusion criteria of the review, including how many reviewers screened each record and each report retrieved, whether they worked independently, and if applicable, details of automation tools used in the process. | 6 |
| Data collection process | 9 | Specify the methods used to collect data from reports, including how many reviewers collected data from each report, whether they worked independently, any processes for obtaining or confirming data from study investigators, and if applicable, details of automation tools used in the process. | 6 |
| Data items | 10a | List and define all outcomes for which data were sought. Specify whether all results that were compatible with each outcome domain in each study were sought (e.g. for all measures, time points, analyses), and if not, the methods used to decide which results to collect. | 6-7 |
|  | 10b | List and define all other variables for which data were sought (e.g. participant and intervention characteristics, funding sources). Describe any assumptions made about any missing or unclear information. | 7-8 |
| Study risk of bias assessment | 11 | Specify the methods used to assess risk of bias in the included studies, including details of the tool(s) used, how many reviewers assessed each study and whether they worked independently, and if applicable, details of automation tools used in the process. | 8-9 |
| Effect measures | 12 | Specify for each outcome the effect measure(s) (e.g. risk ratio, mean difference) used in the synthesis or presentation of results. | 7 |
| Synthesis methods | 13a | Describe the processes used to decide which studies were eligible for each synthesis (e.g. tabulating the study intervention characteristics and comparing against the planned groups for each synthesis (item #5)). | 9 |
|  | 13b | Describe any methods required to prepare the data for presentation or synthesis, such as handling of missing summary statistics, or data conversions. | 7 |
|  | 13c | Describe any methods used to tabulate or visually display results of individual studies and syntheses. | 10 |
|  | 13d | Describe any methods used to synthesize results and provide a rationale for the choice(s). If meta-analysis was performed, describe the model(s), method(s) to identify the presence and extent of statistical heterogeneity, and software package(s) used. | 7-8 |
|  | 13e | Describe any methods used to explore possible causes of heterogeneity among study results (e.g. subgroup analysis, meta-regression). | 9 |
|  | 13f | Describe any sensitivity analyses conducted to assess robustness of the synthesized results. | 9 |
| Reporting bias assessment | 14 | Describe any methods used to assess risk of bias due to missing results in a synthesis (arising from reporting biases). | 8 |
| Certainty assessment | 15 | Describe any methods used to assess certainty (or confidence) in the body of evidence for an outcome. | 8 |
| **RESULTS** | | |  |
| Study selection | 16a | Describe the results of the search and selection process, from the number of records identified in the search to the number of studies included in the review, ideally using a flow diagram. | 9 |
|  | 16b | Cite studies that might appear to meet the inclusion criteria, but which were excluded, and explain why they were excluded. | 5 |
| Study characteristics | 17 | Cite each included study and present its characteristics. | 9-10; 29; S4 |
| Risk of bias in studies | 18 | Present assessments of risk of bias for each included study. | 29; S7-S8 |
| Results of individual studies | 19 | For all outcomes, present, for each study: (a) summary statistics for each group (where appropriate) and (b) an effect estimate and its precision (e.g. confidence/credible interval), ideally using structured tables or plots. | 34 |
| Results of syntheses | 20a | For each synthesis, briefly summarise the characteristics and risk of bias among contributing studies. | 10-11 |
|  | 20b | Present results of all statistical syntheses conducted. If meta-analysis was done, present for each the summary estimate and its precision (e.g. confidence/credible interval) and measures of statistical heterogeneity. If comparing groups, describe the direction of the effect. | 9-10 |
|  | 20c | Present results of all investigations of possible causes of heterogeneity among study results. | 10-12 |
|  | 20d | Present results of all sensitivity analyses conducted to assess the robustness of the synthesized results. | 10-11 |
| Reporting biases | 21 | Present assessments of risk of bias due to missing results (arising from reporting biases) for each synthesis assessed. | 10; S7-S8 |
| Certainty of evidence | 22 | Present assessments of certainty (or confidence) in the body of evidence for each outcome assessed. | 10; S8 |
| **DISCUSSION** | | |  |
| Discussion | 23a | Provide a general interpretation of the results in the context of other evidence. | 13-14; 16-17 |
|  | 23b | Discuss any limitations of the evidence included in the review. | 18 |
|  | 23c | Discuss any limitations of the review processes used. | 18 |
|  | 23d | Discuss implications of the results for practice, policy, and future research. | 18-19 |
| **OTHER INFORMATION** | | |  |
| Registration and protocol | 24a | Provide registration information for the review, including register name and registration number, or state that the review was not registered. | 2,5 |
|  | 24b | Indicate where the review protocol can be accessed, or state that a protocol was not prepared. | 5 |
|  | 24c | Describe and explain any amendments to information provided at registration or in the protocol. | 5 |
| Support | 25 | Describe sources of financial or non-financial support for the review, and the role of the funders or sponsors in the review. | 20 |
| Competing interests | 26 | Declare any competing interests of review authors. | 1 |
| Availability of data, code and other materials | 27 | Report which of the following are publicly available and where they can be found: template data collection forms; data extracted from included studies; data used for all analyses; analytic code; any other materials used in the review. | 5 |

**Table S2.** Further details of the RCT studies contained in the meta-analysis. Acronyms are as follows: ALA: alpha-linolenic acid; ASR: Adult Self-Report (Achenbach & Rescorla 2001); ABCL: Adult Behavior Checklist (Achenbach & Rescorla 2001); CBCL: Child Behavior Checklist (Achenbach & Rescorla 2001); CD: conduct disorder; CODDS: Conduct and Oppositional defiant Disorder Scales (Raine et al. 2022); DISC-IV: NIMH Diagnostic Interview Schedule for Children, Version IV (Schaffer et al., 2000); ODD: oppositional defiant disorder; DHA: docosahexaenoic acid; EPA: eicosapentaenoic acid; SDQ: Strengths and Difficulties Questionnaire (Goodman 1997); SNAP-IV: Swanson, Nolan and Pelham - IV (Parent-reported rating scale) (Swanson 1992).

| **Study** | **Measure of Antisocial Behavior** | **Omega-3** | **Form of delivery** | **Placebo**  **Constituents** | **Active**  **Constituents** |
| --- | --- | --- | --- | --- | --- |
|  |  |  |  |  |  |
|  |  |  |  |  |  |
| Bélanger et al. (2009) | ODD (Connors) | EPA+DHA | Capsule supplement | Sunflower oil | Fish oil |
| Bos et al. (2015) | Rule-Breaking (CBCL) | EPA+DHA | Margarine | Normal margarine | Omega-3 fortified margarine |
| Crippa et al. (2019) | Conduct Problems (SDQ) | DHA | Gelatin pearls | Wheat germ oil | Algae/algal DHA |
| Dean et al. (2014) | Conduct Problems (SDQ) | EPA+DHA | Capsule supplement | Low polyphenol olive oil and 10mg standard fish oil | Fish oil |
| Gesch et al. (2002) | Rule-breaking (Governor Reports^1^) | EPA+DHA+ALA | Capsule supplement | Vegetable oil | Efamol Marine (unable to find origin, likely fish) |
| Gustafsson et al. (2010) | ODD (Connors) | EPA+DHA | Capsule supplement | Rapeseed oil and medium-chain triglycerides | PlusEPA (fish oil) |
| Johnson et al. (2010) | ODD (CBCL) | DHA | Capsule supplement | Olive oil | Algal oil from Martek Biosciences DHA supplement |
| Kean et al. (2017) | ODD and CD (Connors) | EPA+DHA | Capsule supplement | Olive oil, coconut oil, lecithin | New Zealand green-lipped mussel (omega-3 anti-inflammatory extract PCSO-524) |
| Kirby et al. (2010) | Conduct Problems (SDQ) | EPA+DHA | Chewable gelatin capsules | Olive oil | Fish oil |
| Manor et al. (2012) | ODD (Connors) | EPA+DHA | Capsule supplement | Cellulose | Shellfish |
| Milte et al. (2015) | ODD (Connors) | EPA+DHA | Capsule supplement | Safflower oil | Fish oil |
| Raine et al. (2015) | Rule-Breaking (CBCL) | EPA+DHA+ALA | Fruit Juice Drink (Smartfish Recharge) | Fruit juice | Fish oil |
| Raine et al. (2016) | Rule-breaking (CBCL); ODD and CD (CODDS); ODD and CD (DISC-IV) | EPA+DHA+ALA | Fruit Juice Drink (Smartfish Recharge) | Fruit juice | Fish oil |
| Raine et al. (2019) | Rule-breaking (CBCL); ODD and CD (CODDS); ODD (Connors) | EPA+DHA | Capsule supplement | Sunflower oil (with a small amount of fish oil for taste) | Fish oil |
| Raine et al. (2020) | Rule-breaking (ASR and ABCL); ODD  and CD (CODDS); rule-breaking (Governor Reports^1^) | EPA+DHA+ALA | Fruit Juice Drink Drink (Smartfish Recharge) | Fruit juice | Fish oil |
| Raine et al. (2021) | ODD and CD (CODDS) | EPA+DHA+ALA | Fruit Juice Drink (Smartfish Recharge) | Fruit juice | Fish oil |
| Richardson and Puri (2002) | ODD (Connors) | EPA+DHA+ALA | Capsule supplement | Olive oil | Not reported |
| Richardson and Montgomery (2005) | ODD (Connors) | EPA+DHA | Capsule supplement | Olive oil | Fish oil |
| Richardson et al. (2012) | ODD (Connors) | DHA | Capsule supplement | Corn/soybean oil | Algal oil |
| Sinn and Bryan (2007) | ODD (Connors) | EPA+DHA | Capsule supplement | Palm oil | Fish oil |
| Smuts et al. (2015) | ODD (Connors) | EPA+DHA | Capsule supplement | Medium-chain TAG | Fish oil |
| Stevens et al. (2003) | CD and ODD (Pelham DBD Scale) | EPA+DHA+ALA | Capsule supplement | Olive oil | Efalex (fish oil) |
| Tammam et al. (2016) | ODD (Connors) | EPA+DHA | Capsule supplement | Sunflower oil | WellTeen Plus (fish oil) |
| Widenhorn-Muller et al. (2014) | Rule-Breaking (CBCL) | EPA+DHA | Capsule supplement | Olive oil | Fish oil |
| Young et al. (2017) | ODD (SNAP-IV) | EPA+DHA | Capsule supplement | Couldn’t find | OmegaBrite (fish oil) |

^1^ Governor reports in these UK and Singapore studies of young offenders refer to internal disciplinary proceedings concerning breaking of prison rules. In both studies only minor reports were extracted, with typical offences consisting of disobedience, refusing an order, possession of prohibited items, stealing, being found in an unauthorized area.

Moderator Analyses on Antisocial Measures in Table S1.

With respect to whether the form of antisocial measure may moderate treatment outcome, we ran additional moderator analyses. As can be seen in Table S1, 11 studies reported specifically on oppositional defiant behavior, 3 reported specifically on conduct disorder, 4 reported specifically on rule-breaking behavior, while the remaining 6 studies reported on a combination of antisocial outcomes. Results for moderation analyses were as follows: ODD versus CD: F(1,12) = 2.76, p = .12; ODD versus rule-breaking: F(1,12) = 1.07, p = .32, and ODD versus all others: F(1,26 = 1.03, p = .32. As such, there was no significant moderation, although in the light of the small numbers in some sub-groups, these results must be treated with caution and await confirmation from future RCTs that can provide more power to detect differences.

**References for Antisocial Measures in Table S1**

Achenbach, T. M., & Rescorla, L. A. (2001). *Manual for the ASEBA school-age forms & profiles: child behavior checklist for ages 6-18, teacher's report form, youth self-report: an integrated system of multi-informant assessment*. University of Vermont, Research Center for Children Youth & Families.

Conners, C. K., Sitarenios, G., Parker, J. D., & Epstein, J. N. (1998). Revision and restandardization of the Conners Teacher Rating Scale (CTRS-R): factor structure, reliability, and criterion validity. *Journal of Abnormal Child Psychology, 26*(4), 279-291.

Goodman, R. (1997). The Strengths and Difficulties Questionnaire: a research note. *Journal of Child Psychology and Psychiatry, 38*(5), 581-586.

Raine, A., Ling, S., Streicher, W., & Liu, J. (2022). The conduct and oppositional defiant disorder scales (CODDS) for disruptive behaviour disorders. *Psychiatry Research, 316*, 114744.

Shaffer, D., Fisher, P., Lucas, C. P., Dulcan, M. K., & Schwab-Stone, M. E. (2000). NIMH Diagnostic Interview Schedule for Children Version IV (NIMH DISC-IV): Description, differences from previous versions, and reliability of some common diagnoses. *Journal of the American Academy of Child & Adolescent Psychiatry, 39*(1), 28-38.

Swanson, J. M. (1992). *School-based assessments and interventions for ADD students*. Irvine: KC Publishing

**Appendix S1. Study Quality Assessment**

The quality of each of the 25 RCTs in the meta-analysis was evaluated using the 14-item NIH Quality Assessment Tool for Controlled Intervention Studies (National Heart, Lung, and Blood Institute, 2013). Each item was scored as yes (1 point: no risk of bias), no (0 points: risk of bias), or cannot be determined/not reported. As a conservative estimate, scores coded as cannot be determined/not reported were recoded as 0. Before conducting evaluations on the 25 RCTs in the meta-analysis, two raters first independently scored three RCTs (all on omega-3) that were not contained in the meta-analysis, after which scores were compared and consensus reached on discrepancies. Ratings on the 25 RCTs were then conducted independently by the two raters. As per NIH guidelines, an overall quality rating for each study, and also the entire study pool, was assigned by each rater as “good”, “fair”, or “poor”. Following Sum et al. (2022), scores of 10-14 were categorized as “good quality”, 7-9 as “fair”, and 0-6 as “poor”.

Inter-rater reliability between the scores from two independent evaluators of the 25 studies was high (r = .92, p < .001). External validity of the study quality assessment scores derived from consensus was evaluated by evaluating the relationship of the 25 study scores with the impact score of the journal in which they were published. The assumption is that better quality studies are published in journals with higher impact. The correlation was significant and positive, with higher quality scores being associated with a higher impact factor, r = 71, p < .001. Study quality score was unrelated to study effect size, *β* = .009, SE = .021, t = 0.42, p = .68.

The 14 items used in the NIH Quality Assessment Tool for Controlled Intervention Studies have three scoring option: yes, no, or cannot be determined/ not reported/ not applicable. Consensus evaluations on each of the 14 items are given below. Overall, quality was rated as good.

**Figure S1. Consensus evaluations on each of the 14 items in the Quality Assessment Tool.**

.

The 14 Items are as follows:

Q1. Was the study described as randomized, a randomized trial, a randomized clinical trial, or an RCT?

Q2. Was the method of randomization adequate (i.e., use of randomly generated assignment)?

Q3. Was the treatment allocation concealed (so that assignments could not be predicted)?

Q4. Were study participants and providers blinded to treatment group assignment?

Q5. Were the people assessing the outcomes blinded to the participants' group assignments?

Q6. Were the groups similar at baseline on important characteristics that could affect outcomes (e.g., demographics, risk factors, co-morbid conditions)?

Q7. Was the overall drop-out rate from the study at endpoint 20% or lower of the number allocated to treatment?

Q8. Was the differential drop-out rate (between treatment groups) at endpoint 15 percentage points or lower?

Q9. Was there high adherence to the intervention protocols for each treatment group?

Q10. Were other interventions avoided or similar in the groups (e.g., similar background treatments)?

Q11. Were outcomes assessed using valid and reliable measures, implemented consistently across all study participants?

Q12. Did the authors report that the sample size was sufficiently large to be able to detect a

difference in the main outcome between groups with at least 80% power?

Q13. Were outcomes reported or subgroups analyzed prespecified (i.e., identified before analyses were conducted)?

Q14. Were all randomized participants analyzed in the group to which they were originally assigned, i.e., did they use an intention-to-treat analysis?

National Heart, Lung, and Blood Institute (2013). Study quality assessment tools. Bethesda: U.S. Department of Health & Human Services. <https://www.nhlbi.nih.gov/health-topics/study-qualityassessment-tools> Accessed August 11, 2025.

Sum, G., Nicholas, S.O., Nai, Z.L. et al. (2022). Health outcomes and implementation barriers and facilitators of comprehensive geriatric assessment in community settings: a systematic integrative review [PROSPERO registration no.: CRD42021229953]. *BMC Geriatr.*, *22*, 379. <https://doi.org/10.1186/s12877-022-03024-4>

**Figure S2.** Forest plot of the 19 independent laboratories. Blue boxes indicate the effect size for each study along with the confidence interval (horizontal lines). The red diamond indicates the overall effect size (g = .162) along with its confidence interval. A positive effect indicates that omega-3 supplementation is associated with reduced antisocial behavior.

**Figure S3**. Funnel plot of the effect sizes drawn from the 19 independent laboratories (blue circles), with one imputed study (red dot). Dotted lines indicate pseudo confidence intervals.
